# Intrathyroidal parathyroid carcinoma: a case report with clinical and histological findings

**DOI:** 10.1186/1746-1596-3-46

**Published:** 2008-11-25

**Authors:** Labiba Temmim, Fred Sinowatz, Wiam I Hussein, Osama Al-Sanea, Hady El-Khodary

**Affiliations:** 1 Department of Pathology & Laboratory Medicine Saad Specialist Hospital, Al-Khobar Kingdom of Saudi Arabia; 2 Institute of Histology & Embryology, University of Munich, Germany; 3 Section of Endocrinology Saad Specialist Hospital, Al-Khobar Kingdom of Saudi Arabia; 4 Section of Endocrine Surgery, Saad Specialist Hospital, Al-Khobar Kingdom of Saudi Arabia

## Abstract

The chance of an intrathyroidal occurrence of a parathyroid gland is about 1–3%. Among the causes of hyperparathyroidism, parathyroid cases occur in less than 1% of patients. Here we present the case of a 63 year old Saudi female suffering from an intrathryroidal parathyroid carcinoma. The suspicion coming from the clinical investigations that the removed tumor tissue may be a parathyroid carcinoma could be confirmed by histology. Additionally non-radioactive in situ hybridization to localize mRNA transcripts for Cyclin D1 and immunohistochemical localization of Cyclin D1 was performed. Although parathyroid adenoma and carcinoma have disparate natural history, it can be difficult to differentiate between the two entities. Clinical presentation, operative findings may raise suspicion, but may not be conclusive especially if there is no evidence of invasion or metastasis, especially if the gland was intrathyroidal.

## Background

Parathyroid adenomas account for 85% of primary hyperparathyroidism [1]. On the other hand, parathyroid carcinoma is a rare disease that accounts only for 1% to 3% of cases of primary hyperparathyroidism [2,3] and to the best of our knowledge, intrathyroidal parathyroid carcinoma have been reported only three times [3]. The diagnosis and appropriate treatment of intrathyroidal parathyroid carcinoma is difficult and its treatment is more challenging to the surgeons. Adenomas and carcinomas of the parathyroid gland have disparate natural histories, but it can be difficult to differentiate them on the basis of clinical and histological findings alone. Thick fibrous bands, mitotic activity, trabecular growth pattern and capsular, vascular, and adjacent soft tissue invasion have been considered characteristic of parathyroid carcinoma, but some of these morphological features (fibrous bands, mitotic activity, trabecular growth) have been identified in parathyroid adenomas as well [4]. Clarification of the molecular pathogenesis of parathyroid carcinoma can aid in diagnostically difficult cases and may provide important clues for a more effective therapy. Cyclin D1 (CD1) or PRAD1 is a protoncogene located at chromosome band 11q13 and its protein product is a cell cycle regulator [5]. Cyclin D1 gene amplification has been implicated in the pathogenesis of numerous tumors [5-7]. Recently, Hsi et al. [6] demonstrated the over expression of the cyclin D1 oncogene in 18% of 65 patients with parathyroid tumors. They think that over expressed cyclin D1 plays a role in the pathogenesis of a much larger proportion of parathyroid adenomas than previously assumed. Cyclin D1 overexpression is a feature of typical parathyroid adenomas as had been suggested by early DNA studies [7]. Although His et al studied only three patients with parathyroid carcinoma, two of the patients tumors stained strongly for Cyclin D1, raising the possibility that the frequency of Cyclin D1 overexpression may be greater in carcinoma. Cyclin D1 overexpression appears to highlight a central pathway in parathyroid neoplasia. In our case study, we therefore include the localization of Cyclin D1 at the messenger RNA (in situ hybridization) and protein (immunohistochemistry) level.

## Case presentation

**Figure 1:**
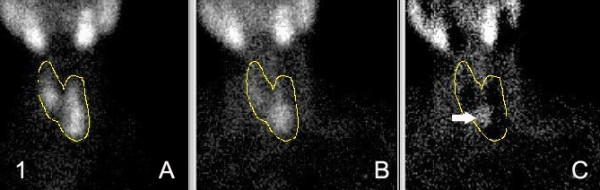
Parathyroid carcinoma occupying nearly the whole thyroid gland (Th). Thick sharply outlined bands of acellular collagenous tissue completely divide the tumor into irregular lobules. The tumor cells are arranged in diffuse masses, solid sheets, closely packed nests or compact trabeculae (H&E). Bar: 250 μm.

A 63-year-old Saudi female, who was known to have hypertension and hyperlipidaemia, complained of fatigue, bone and muscle pain of several years of duration. She also had a history of recurrent nephrolithiasis. Her symptoms aggravated recently with several attacks of abdominal pain, nausea and vomiting. Neck examination revealed a left lower cervical mass, consistent with thyroid enlargement. Ultrasonography showed an enlarged left lobe with multiple hypoechoid and isoechoid nodules. One of them in the anterior-inferior aspect of the left lobe displayed a rim of calcification. Thyroid function test were within the normal range. Parathyroid imaging scan using 99m Tc-SestaMIBI-99c-Pertechnetate was consistent with scintigraphic features of a parathyroid adenoma (Figure 1) in the region of left lower parathyroid gland. X-ray showed a "salt and pepper" skull and diffuse osteopenia. The fine needle aspiration (FNA) of the cervical mass was performed to evaluate left thyroid pathology and showed findings consistent with parathyroid neoplasm.

The patient was admitted and loaded with intravenous saline followed by Furosemide (10 mg) the day before surgery, which was able to bring the serum calcium to 3.59 on the day of surgery. The patient was taken to the operating room where a video-assisted exploration of the left neck was attempted. The surgeon's plan was to remove the diseased parathyroid gland and to take out the enlarged left hemithyroid, followed by frozen section evaluation. Total thyroidectomy is only performed in our clinic, if frozen sections yield evidence of a differentiated thyroid carcinoma greater than one centimeter in diameter. A normal lower left parathyroid gland was identified in the left thymic horn and preserved. The left thyroid lobe appeared enlarged and showed only one distinct nodule near the upper pole, which was sent for frozen section diagnosis. It turned out to be benign thyroid tissue. The surgeon decided then to remove the left thyroid lobe after full neck exploration including the superior mediastinum that revealed four benign central neck lymph nodes.

**Figure 2:**
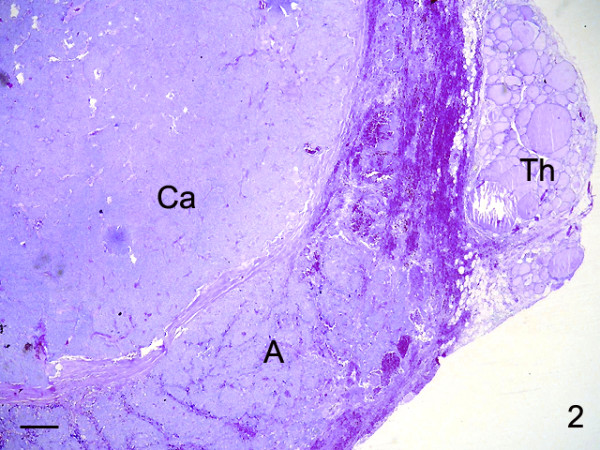
99m Tc SestaMIBI "A" nodular left lobe with sizeable lower pole. "B" prominent focal area in the lower pole of the left lobe of the thyroid. "C" substraction image show the prominent hot area (arrow) to be located in the hot area "A".

The pathology report described a thyroid lobe with a large tumor mass, measuring 6 × 5 cm and weighing 8.5 g. It appeared poorly circumscribed and of hard consistence. The mass showed a gray-whitish color with occasional foci of necrosis. A firm fibrous capsule surrounded it. The tumor occupied nearly the whole thyroid gland. Histology showed that thick, sharply outlined bands of collagenous tissue extended from the thickened capsule into the interior and completely divided the tumor into irregular lobules. The tumor cells were arranged in diffuse masses, solid sheets or closely packed nests of cells (Figure 2). The tumor cells mostly showed round to ovoid, markedly enlarged nuclei with prominent nucleoli and clearly demarcated cytoplasm. Parts of the tumor consisted of cells with abundant, clear to eosinophillic cytoplasm, nuclear pleomorphism, marked cellular atypia. Occasionally macronucleoli and multinucleated giant cells were found. Mitoses were difficult to detect (2 per 10 high power fields). Scattered foci of necrosis and hemorrhage occurred at the periphery of the tumor. Capsular and vascular invasion with tumor cells was observed. Extra-capsular infiltration into the adjacent soft tissue and skeletal structures was also found. The tumor obviously had destroyed most of thyroid gland, leaving only a thin rim of normal thyroid tissue at the periphery (Figure 2). None of the four lymph nodes draining the suspicious left parathyroid gland showed tumor involvement.

Clarification of the molecular pathogenesis of parathyroid carcinoma can aid in diagnostically difficult cases and may provide important clues for a more effective therapy. Therefore, tumor samples (one with a histologically verified parathyroid carcinoma and four additional specimen with histologically diagnosed parathyroid adenoma) were studied using in situ hybridization and immunocytochemistry with monoclonal antibodies. Formalin-fixed (3.7%), paraffin-embedded samples were used to localize mRNA transcripts for Cyclin D1. In situ hybridisation was performed as previously described. The sequences of the oligonucleotides were designed using the program Primer3 . The sequences of the antisense oligonucleotides for Cyclin D1 was as follows: 5'gtt cct cgc aga cct cca gca tcc ag3' Acc.Nr: NM_053056 (NCBI). Immunohistochemical staining was performed with monoclonal anti-Cyclin D1 anti-body (Novocastra/Vector, Burlingame, CA, USA), at dilution of 1:100, using a standard avidin/biotin complex (ABC) method. Breast cancer cases known to express Cyclin D1 served as positive control for Cyclin D1. For negative control, non-immune serum was substituted for primary antibody.

**Figure 3:**
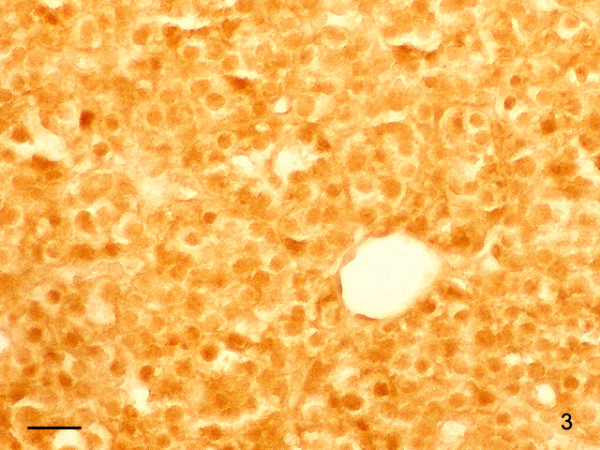
In situ hybridization of Cyclin D1 in a parathyroid adenoma. Cytoplasm and nuclei contain a slight to distinct signal for CD1 mRNA. Bar: 100 μm.

**Figure 4:**
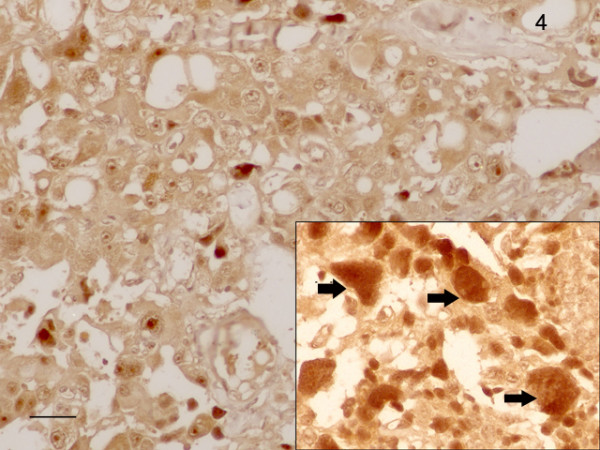
An interesting pattern of CD1-mRNA distribution was seen in the carcinomatous areas, where strongly staining tumor cells varied with nearly CD1-negative ones. The inset shows giant cells distinctly positive for CD1-mRNA. Bar: 100 μm.

**Figure 5:**
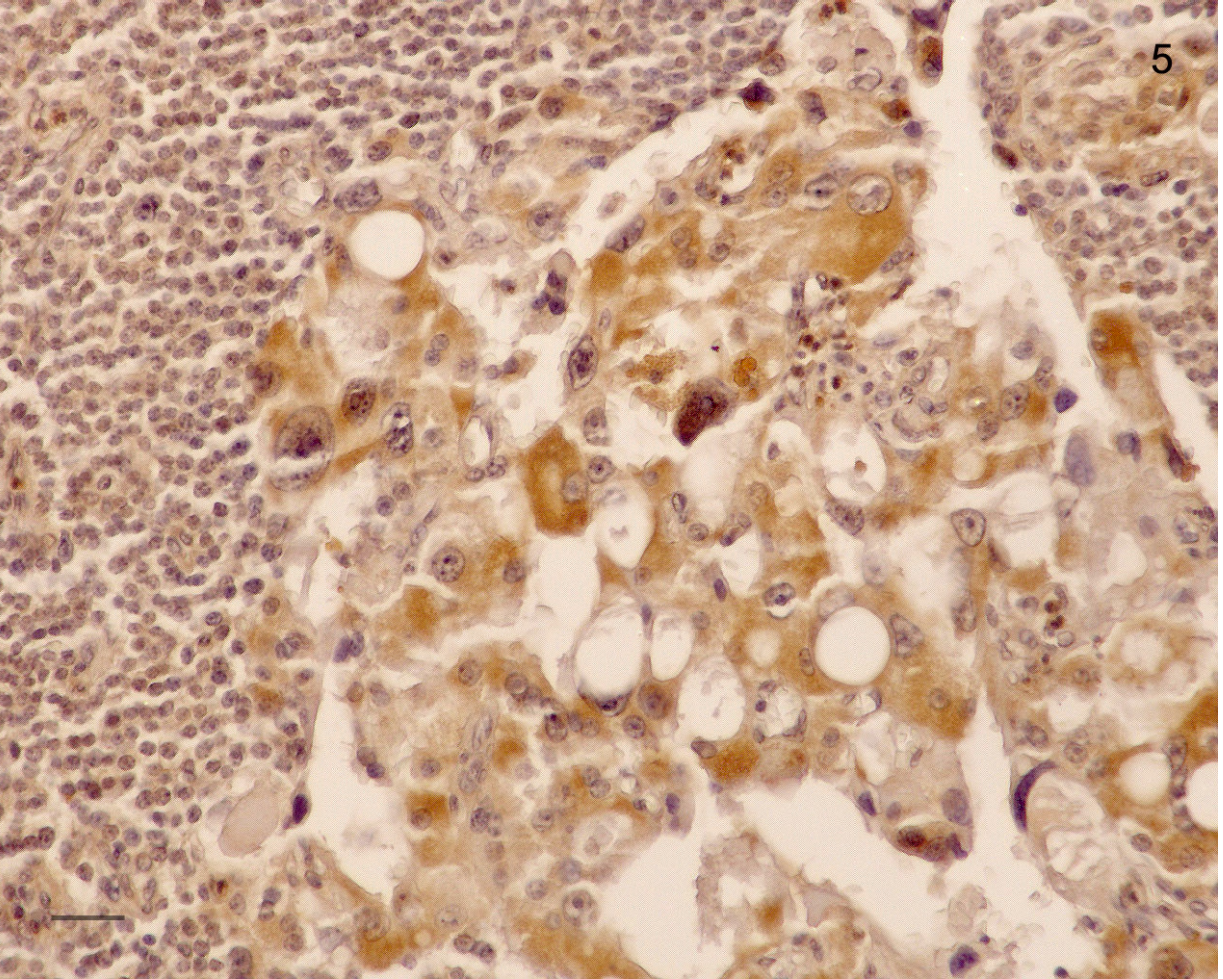
Immunohistochemical localization of CD1 in parathyroid adenoma (A) and parathyroid carcinoma (Ca). Whereas the adenoma cells generally showed a weak to moderate staining, parathyroid carcinoma cells displayed a variable immunostaining for CD1: carcinoma cells strongly positive from CD1 varied with only weakly stained tumor cells. Th: Thyroid tissue. Bar: 100 μm.

Using in situ hybridization we found a weak to distinct signal for CD1 in the cytoplasm of glandular cells of the parathyroid adenoma tissue (Figure 3). No staining was seen in the fibrous stroma that occasional formed broad bands in the neoplasm. Parathyroid carcinoma cells showed a variable staining with the probe used: Cells strongly positive from CD1 mRNA varied with only weakly stained tumor cells. (Figure 4). The giant cells always displayed a strong signal for CD1-mRNA. All 4 adenoma samples showed a weak to distinct immunostaining for the CD1 protein in their cytoplasm. In about 20% of the cells, also a nuclear staining occurred. The corresponding stroma in the adenomas showed no immunoreactivity for CD1. Also with immunohistochemistry, parathyroid carcinoma cells showed a variable staining for CD1 and carcinoma cells strongly positive from CD1 mRNA varied with only weakly stained tumor cells (Figure 5). Therefore, the most distinct feature distinguishing parathyroid adenoma from parathyroid carcinoma cells is the occurrence of strongly CD1 positive cells in the carcinoma areas.

At the postoperative patient visit, a week after surgery, the serum calcium has dropped to 2.38 mg/dl (2.10–2.55), the parathormone (PTH) level has returned to normal values, however alkaline phosphatase remained high (628 U/L). CT scan of the brain and lungs revealed no evidence of metastasis. At follow-up three months after surgery, the patient appeared disease free with normal levels of PTH and calcaemia and improvement of the bone disease symptoms. The patient is now, two years from surgery, disease free and her serum calcium and parathormone level remained in the normal range.

## Conclusion

Parathyroid carcinoma is a rare malignancy with an incidence of less than 1% in all patients surgically treated for primary hypoparathyroidism [8-12]. A higher rate of about 5% has only reported in few countries, like Japan and Italy [13]. Most reports indicate equal gender distribution and a mean age of 48 years, a decade earlier than adenoma. The clinical manifestations of hyperparathyroidism in parathyroid carcinoma are usually more severe than in patients with parathyroid adenoma with markedly elevated serum calcium levels (and resulting symptoms like muscle weakness, fatigue, depression, nausea, polyuria) and parathormone (PTH) levels [14,15]. In our case PTH level was more phosphatase was also significantly higher.

The diagnosis of malignancy was made post operatively. The fine needle aspiration performed intra operatively was done to evaluate the left thyroid lobe lesion rather and revealed normal thyroid tissue. None of the preoperative studies raised suspicion of the presence of parathyroid malignancy and the probability to find a parathyroid carcinoma in patients with palpable parathyroid glands, marked elevation of serum calcium and parathormone [14,15] is less than 5%. Due to the intrathyroidal location [16-18], there was no strong indication of malignancy in this case at the beginning. Severe symptoms of renal disease (impaired renal function, nephrolithiasis, nephrocalcinosis) are also requent in patients with benign parathyroid tumors. Bone pain, "salt and pepper " skull and diffuse osteopenia were the bone symptoms present in our patient. The combination of both renal and bone symptomes at presentation and the presence of a palpable neck mass raised the suspicion of malignancy [16].

The suspicion that the removed tumor tissue may be a parathyroid carcinoma could be confirmed by histology. In our case, invasion of tumor cells was found in the vessels and neighboring muscle tissue, which has been seen also in other studies [3,10]. Cellular atypia and variation, also seen in the tumor samples of our case, are not particular useful criteria to diagnose a parathyroid carcinoma, as no specific morphologic criteria exist. In 1993, Bondeson et al. [9] reported that parathyroid carcinoma may have such bland cytologic appearance that nothing but the invasiveness of the tumor can differentiate them from a benign lesion, although fibrosis, necrosis, nuclear atypia (pleomorphism macronucleoli, large nuclei) and mitotic figures are significantly more frequent in carcinoma. Capsular invasion is present in 60% of the parathyroid carcinomas. They affect the surrounding skeletal muscle, thyroid gland [17,18] or other tissues. Vascular invasion is characterized by carcinoma cells present within vessels of the fibrous tumor capsule. This is not a very common finding in carcinoma, but was present in our case and can be interpretated as clear sign of malignancy [10]. Solid growth of the parathyroid carcinoma with tumor cells arranged in diffuse masses, small nests or trabeculae are typically found [10]. All these histological features were found in our case. In other cases of parathyroid carcinomas follicular or spindle cell patterns or, rarely, papillary growth has been reported [1,10].

A change in the expression of CD1 mRNA has been found during the progression from benign to malignant parathyroid tumor cells CD1 oncoprotein is overexpressed frequently in parathyroid carcinoma [5-7]. It was proposed that s could be used as some additional diagnostic criteria [7]. In our case, the only clearly seen difference in the expression of CD1 in benign versus malign parathyroid tissue was the occurrence of strongly CD1 positive giant cells within the carcinoma. Using immunocytochemistry, these cells are easily found and could support the pathohistological diagnosis.

In summary, parathyroid carcinoma is a rare malignancy. Signs and symptoms of hypocalcaemia, hyperparathyroid bone disease and features of renal involvement along with a palpable neck mass are strong predictors of parathyroid malignancy. It is usually difficult to recognize the tumor preoperatively and it is not conclusively identified intraoperatively. Pathologic histology including in situ hybridization and immunohistochemistry of cyclin D1 helps to characterize the lesion and confirms its nature.

## Consent

The patient has given her consent for the case report to be published.

## Competing interests

The authors declare that they have no competing interests.

## Authors' contributions

LT, and FS drafted the manuscript. WIH and OAS compiled the clinical data. LT and HEK confirmed the clinical diagnosis by histopathology. FS evaluated the immunohistochemical stainings and non radioactive in situ hybridization. All authors read and approved the final manuscript
